# Evaluation of the Vegetation Coverage Resilience in Areas Damaged by the Wenchuan Earthquake Based on MODIS-EVI Data

**DOI:** 10.3390/s17020259

**Published:** 2017-01-28

**Authors:** Xiaofu Liu, Weiguo Jiang, Jing Li, Wenjie Wang

**Affiliations:** 1 State Key Laboratory of Earth Surface Processes and Resource Ecology, Beijing Normal University, Beijing 100875, China; liuxf@craes.org.cn (X.L.); lijing@bnu.edu.cn (J.L.); 2 Institute of Environmental Information, Chinese Research Academy of Environmental Sciences, Beijing 100012, China; wangwj@craes.org.cn; 3 Academy of Disaster Reduction and Emergency Management, Beijing Normal University, Beijing 100875, China

**Keywords:** ecological resilience, MODIS-EVI, vegetation cover, earthquake, recovery interval

## Abstract

The concept of resilience was integrated into post-earthquake ecological restoration assessments in 10 counties heavily impacted by the 2008 Wenchuan earthquake. Ecological resilience was defined as the time interval required for the vegetation coverage to recover to pre-earthquake levels in damaged areas. MODIS-EVI data from May to August in 2000 to 2016 were used to calculate the ecological resilience by fitting the curve of recovery rate (RR) versus time. The following conclusions were reached: (1) An area of 424.1 km^2^ sustained vegetation damage. (2) The vegetation recovery was found to be linear based on the statistical analysis of the most common components of the damaged areas; consequently, linear fitting was used to estimate the resilience. (3) In terms of vegetation coverage, 44.2% of the damaged areas have already recovered. The vast majority of damaged areas are predicted to achieve vegetation recovery by 2022, but 5.3% of the damaged areas will not recover within this time period and have no resilience. (4) The management of damaged areas near roads, rivers and mining operations, especially at elevations of 2000–2500 m, slopes greater than 30°, and precipitation levels greater than 1200 mm, should be prioritized in the future. (5) The innovations of this study include the method used to extract earthquake-related vegetation damage and the prediction of vegetation succession based on resilience.

## 1. Introduction

The concept of ecological resilience was first introduced into ecology from physics in 1973 by Holling [1,2], who proposed the concept and defined resilience as the ability of a system to absorb state variables, driving variables and parametric variation without system transitions or qualitative changes [1,2]. In 1984, Pimm defined resilience as the system recovery speed and as the time required to recover to the original state after interference [3]. Based on the two initial definitions, later researchers continued to enrich and improve the resilience concept [4,5]. This concept represents both a resource management approach and a worldview [6,7,8,9,10]. The use of resilience in ecosystem management has become a focus of research [4,11] due to its ability to predict the ecosystem consequences of succession [12]. Unfortunately, regardless of the definition, resilience is difficult to measure [13,14,15,16,17,18]. However, it is certainly easier to develop empirical studies to measure Pimm resilience than Holling resilience [13,19]. Holling resilience is too extensive to define interference [20] and constructing the response relationships between interference and the parameters is difficult [21,22,23]. Under Pimm’s framework, empirical resilience studies have been performed through the comparison of the system before and after interference or through the comparison of disturbed and undisturbed systems, especially in terms of vegetation cover, species diversity, plant ecophysiology, among other factors. Fuzzy evaluation was the most common method for resilience measurements and was often used to assess the potential of vegetation recovery [24,25]. Another method for evaluating resilience was fitting trends in the vegetation coverage evolution [26,27,28]. Based on the long-term time series data of AVHRR-NDVI, system resilience was measured by comparing the decay times of the numbers of pixels that maintained positive and negative tendencies by Lanfredi [26] and later researchers [27,28]. When the decay time of pixels maintaining positive tendencies was less than that of pixels maintaining negative tendencies, the resilience of the vegetation cover was higher; when the opposite was true, the resilience was lower [26]. The slope of the inter-annual variability in carbon sequestration and transpiration was also used to evaluate the ecological resilience following a fire disaster by Vitale [29]. Regardless of the method, i.e., fuzzy evaluation, fitting the vegetation coverage trend or fitting the slope of the inter-annual variability, the ecological resilience results are always dimensionless, which is useful for comparing resilience estimates. Obtaining absolute value estimates for ecosystem resilience is a challenge for future studies.

The Wenchuan earthquake in 2008 not only caused considerable losses of life and property but also produced enormous ecological destruction. Vegetation coverage decreased considerably due to secondary geological disasters, such as collapse events, landslides and mudslides [30]. In recent years, some researchers have attempted to study post-disaster ecosystem recovery via remote sensing, but the results have varied [31,32,33,34,35,36]. Zhang et al. found that the speed of natural recovery was fast and that the slope and soil type were the key factors for vegetation recovery [34]. Hou et al. concluded that the vegetation rapidly recovered between 2009 and 2011 and that the recovery was more obvious in the areas with seismic intensities of X and XI [32]. However, using MODIS-NDVI data to study the variation in vegetation cover after the earthquake, Jiang et al. concluded that the rate of vegetation recovery was relatively slow in the damaged areas and that only 40% of the damaged vegetation area had recovered to the pre-earthquake level by 2013 [35]. At present, research on vegetation recovery has mostly focused on the description of the current recovery level and has lacked predictions of the future trends. We have attempted to introduce resilience into an ecological restoration assessment to demonstrate the current state of vegetation recovery in a damage area and to predict the vegetation evolution in the future. We hope to provide decision support for disaster prevention and ecosystem management.

## 2. Study Area

**Figure 1:**
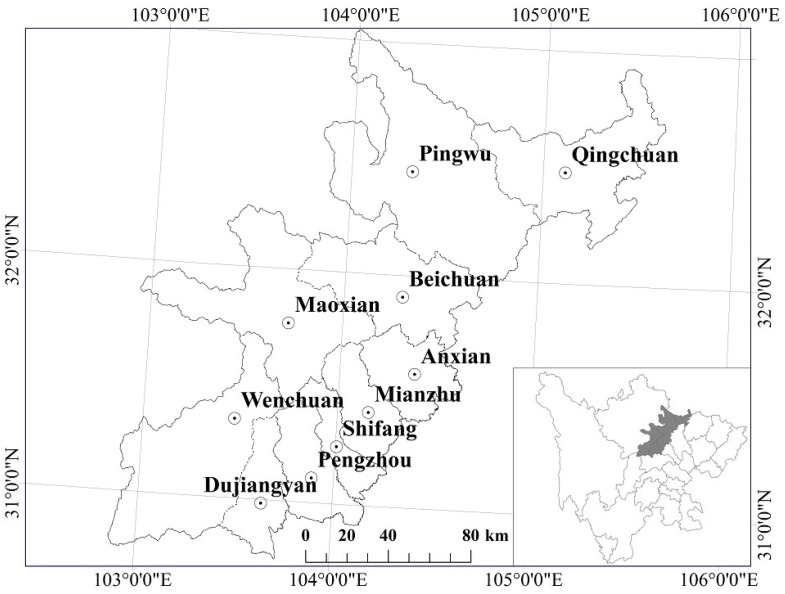
Study area.

This study focused on 10 counties severely affected by the disaster, per the assessment by the Ministry of Land and Resources of People’s Republic of China, namely Wenchuan, Beichuan, Mianzhu, Shifang, Qingchuan, Maoxian, Anxian, Dujiangyan, Pingwu and Pengzhou (Figure 1). The research area featured elevations from 490–5600 m and average annual precipitation values of 490 to 1400 mm, with most areas receiving more than 1000 mm. Some hot dry valleys affected by the Foehn wind in Wenchuan and Maoxian featured little precipitation (500 mm/a) and high evaporation (1500 mm/a). The vertical vegetation zonation was distinct. Below 1000 m, the climate was warm, and vegetation mainly grew on montanic yellow soil and yellow brown soil and was dominated by evergreen species, such as *Lauraceae, Symplocaceae, Theaceae, Fagaceae, Magnoliaceae*, etc. From 1000 m to 3000 m, the climate was cool, humid, and foggy, with limited sunshine, and the vegetation was dominated by evergreen broad-leaf forest and dark coniferous forest. From 3000 m to 3500 m, the climate was cold but with ample sunshine, and the vegetation was mainly coniferous mixed forest dominated by *fir, spruce* and *Juniperus squamata*. Above 3500 m, the climate was extremely cold, dry and windy, and the soil was thin with lots of bare rocks. In this region, the vegetation consisted of alpine bushes and meadows dominated by *Gramineae* and *Cyperaceae*.

## 3. Data Source

The normalized difference vegetation index (NDVI) and enhanced vegetation index (EVI) are commonly used to describe vegetation coverage. In our study area, areas with high vegetation coverage would generate saturated NDVI values [37]; thus, the EVI was used to measure the vegetation coverage in this research. The EVI reduced the variation in the canopy background structure while retaining sensitivity to vegetation density [38]. The MODIS instrument aboard the Terra satellite produced a vegetation index dataset (including NDVI and EVI) known as MYD3Q1 with a temporal resolution of 16 days and a spatial resolution of 250 m. A total of 136 EVI measurements were collected during May to August from 2000 to 2016.

To further understand the spatial variations in resilience and to validate the results, the following data were also adopted: a digital elevation map (1:250,000), average annual precipitation (generated by spatial interpolation of 70 points in the research area), average annual evaporation (generated by the spatial interpolation of 20 points in the research area), land use (1:10,000), soil type (1:1,000,000), lithology (1:500,000), field survey points (gathered by geological experts shortly after the earthquake), Landsat TM images (2007/9/18, 2008/7/19, path/row: 130/38), and Landsat ETM images (2007/5/7, 2008/10/24, path/row: 129/38).

## 4. Methods

### 4.1. The Maximum Value Composite

Annual EVI values were obtained by maximizing the EVI values of May to August (Equation (1)). The maximum value composite not only represented the best growth of vegetation but also reduced the deviation caused by clouds and fog [32,35,39]:

(1) $$EVI_{t}=MAX\left(EVI_{129},EVI_{145},EVI_{161},EVI_{177},EVI_{193},EVI_{209},EVI_{241}\right)$$

where $EVI_{t}$ represents the vegetation coverage of the year *t*. $EVI_{129}$ corresponds to the data of the 129th day in year *t* (the first data point in May), and, based on the 16-day data interval, $EVI_{241}$ corresponds to the second data point in August.

### 4.2. Identification of the Damaged Area

A decline in EVI values could be caused by a sudden earthquake or by gradual changes in natural conditions. When extracting a damaged area, the pixels with EVI values that decreased due to natural changes should be eliminated. Extreme value differences before the earthquake were used to assess the maximum natural fluctuation in the EVI values. Only the pixels in which the decrease in EVI values exceeded the maximum natural fluctuation were extracted to represent the damaged area.

First, the maximum natural fluctuation before the earthquake for the EVI values of each pixel ($EVI_{R}$) was calculated as the difference between the maximum and minimum EVI values from 2000 to 2007 (Equation (2)). Second, the average EVI value of each pixel for the period 2000–2007 ($\bar{EVI_{be}}$) was calculated (Equation (3)) to determine the general level of vegetation coverage before the earthquake. Third, the change in EVI values after the earthquake ($EVI_{C}$) was calculated based on the difference between the EVI values in 2008 and the average value before the disaster (Equation (4)). We then extracted the pixels in which the EVI decrease following the earthquake was greater than the maximum natural fluctuation ($EVI_{C}<0\;\mathrm{and}\;\left|EVI_{C}\right|>EVI_{R}$) (Equation (5)):

(2) $$EVI_{R}=MAX\left(U_{t=2000}^{2007}EVI_{t}\right)-MIN\left(U_{t=2000}^{2007}EVI_{t}\right)$$

(3) $$\bar{EVI_{be}}=\sum_{t=2000}^{2007}EVI_{t}/8$$

(4) $$EVI_{C}=EVI_{2008}-\bar{EVI_{be}}$$

(5) $$D=\left\{EVI|EVI_{C}<0\&\left|EVI_{C}>EVI_{R}\right|\right\}$$

### 4.3. Definition and Measurement of Resilience

In the literature, resilience is considered to represent the ability to rebound and recover. Therefore, because this research focused on vegetation restoration capacity, resilience was defined as the time interval required for the vegetation coverage in the earthquake-damaged area to attain or exceed the level present before the earthquake. The absolute recovery rate, calculated via Equation (6), has commonly been used by previous researchers to evaluate whether vegetation cover has reached the pre-earthquake state [35,40,41]:

(6) $$RR_{t}=(EVI_{t}-EVI_{2008})/\left(\bar{EVI_{be}}-EVI_{2008}\right),\left(t\geq 2009\right)$$

The rapidity of the vegetation recovery process may lead to misleading results when the absolute recovery rate is used [34]. We intended to consider changes in the recovery rate over time and to fit the trend. Thus, the relative recovery rate of each year, calculated via Equation (7), was used to identify whether vegetation coverage recovered to the pre-earthquake state in this research:

(7) $$RR{'}_{t}=EVI_{t}/\bar{EVI_{be}},\left(t\geq 2009\right)$$

where $RR_{t}$ corresponds to the vegetation recovery rate (RR) in year *t* after the earthquake. When $RR_{t}$ ≥ 1, the vegetation coverage has reached or exceeded the pre-earthquake level in year *t*; when 0 ≤ $RR_{t}$ < 1, it has not reached the average pre-earthquake level.

The resilience of the vegetation coverage was represented by the recovery time interval *T*, which was calculated using Equation (8):

(8) $$T=t^{\prime }-2008$$

The term $t^{\prime }$ represents the first year in which $RR_{t}=1$ following the earthquake. To better estimate the recovery time $t^{\prime }$ of each pixel, we set $RR_{t}=1$ when $RR_{t}$ > 1. Two scenarios were considered to calculate the recovery time. Scenario 1: when $RR_{t}=1$ was found in two or more consecutive years in the period 2009–2016, the damaged area had stably recovered to the pre-earthquake level during the evaluation period, i.e.,

(9) $$while\;RR_{t}=1\&RR_{t+1}=1,get\;t^{\prime }=t$$

Scenario 2: when $RR_{t}=1$ was not found in two or more consecutive years from 2009 to 2016, a curve was fitted to the $RR_{t}$ value of each pixel over time. The damaged vegetation area had a high resilience, especially immediately after the earthquake [34]. Hou et al. indicated that, although the vegetation restoration patterns in distinct regions varied, the vegetation evolution trend was linear, significant, and positive after the earthquake [34]. Jiang et al. drew curves for the NDVI mean values in fully, slightly and poorly recovered areas with time from 2009 to 2013 and found somewhat linear relationships [35]. Yang and Qi performed linear regression of the post-seismic vegetation trends and found that 59% of the MODIS-derived damaged vegetation areas showed a decreasing difference between pre- and post-seismic vegetation conditions [42]. The most common components of the annual EVI and RR were extracted based on normal distributions. The statistical properties of the inter-annual variations in the most common components of the EVI and RR were observed to identify the overall vegetation evolution trends. We assumed that the trend was linear, and linear fitting of the RR with time was used to calculate the recovery time. If the trend was obviously nonlinear, other fitting methods would have been considered:

(10) $$RR_{t}=at+b$$

(11) $$Set\;RR_{t}=1,\;get\;t^{\prime }=\left(1-b\right)/a.$$

where *a* and *b* are the slope and intercept, respectively, in the linear equation for each pixel.

When T≤0, the pixels had no resilience (the vegetation could not recover to the pre-earthquake level); when 0<T≤50, the pixels had a certain degree of resilience (the vegetation could recover within 50 years); and when T>50, the pixels had a very low resilience (the vegetation had difficulty recovering to the pre-earthquake level). The recovery interval could be considered “infinite” when T ≤0 and could be considered greater than 50 years when  T>50.

## 5. Results

### 5.1. Dynamics of the Vegetation Coverage in the Damaged Areas

**Figure 2:**
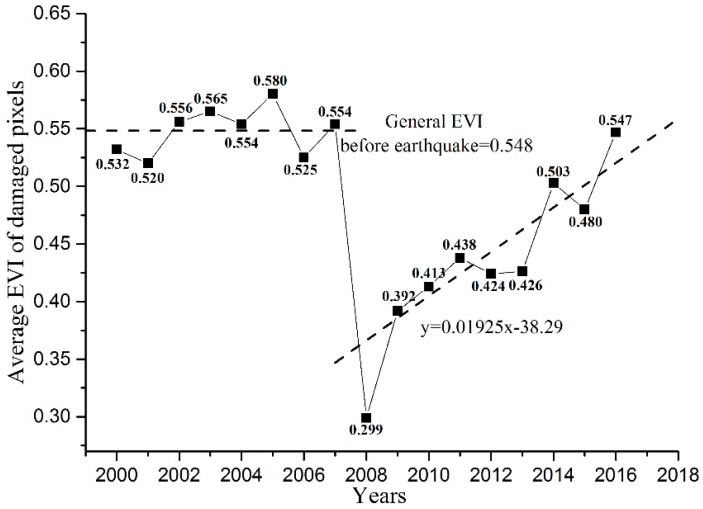
Annual vegetation coverage values in the damaged areas.

The total numbers of pixels and damaged pixels were 485,668 and 7902, respectively. The damaged area covered 424.1 km^2^, representing 1.6% of the entire study area. During 2000–2007, the annual EVI value of the damaged pixels varied between 0.520 and 0.580. The average EVI value of the damaged pixels was 0.548 before the earthquake and dropped markedly to 0.299 after the earthquake in 2008, representing a decline of 45.4%. After 2009, the EVI increased, reaching 0.547 by 2016, demonstrating that the damaged areas generally recovered to the pre-earthquake level (Figure 2).

### 5.2. Spatial Distribution of the Damaged Areas

**Figure 3:**
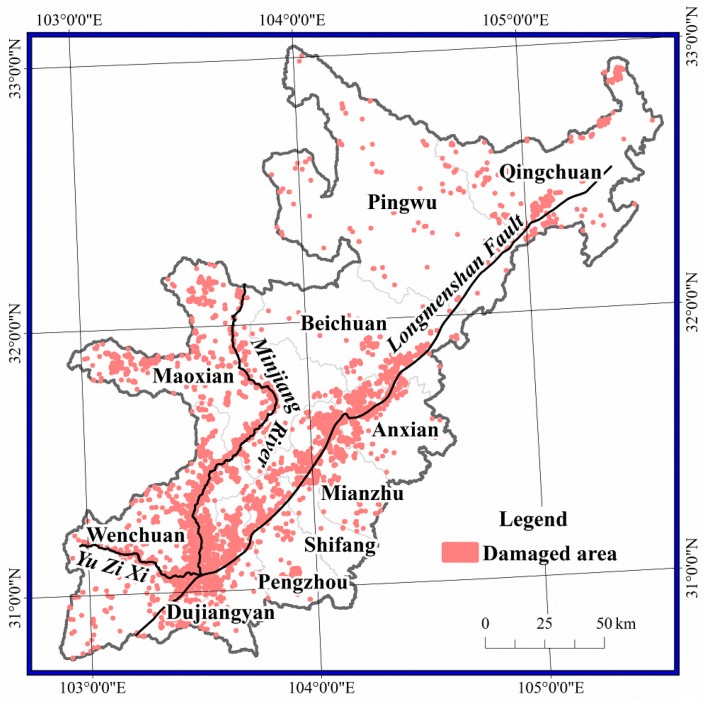
Spatial distribution of damaged pixels.

The damaged areas occurred along three main directions, with Yingxiu at the centre: along the Minjiang River and its tributaries and the G213 National Road to Maoxian; along the Yuzi river and the S303 provincial road to Wolong; and along the Longmenshan fault to Qingchuan (Figure 3). Among the 10 studied counties, the damaged area in Wenchuan was the largest with an area of 183.9 km^2^, representing 43.4% of the total area of damage. The damaged area in Maoxian was the second largest with an area of 52.3 km^2^, representing 12.3% of the total area of damage. The damaged areas in Anxian, Dujiangyan, Mianzhu, Beichuan, Pengzhou, Qingchuan, Shifang and Pingwu were 39.6 km^2^, 36.0 km^2^, 32.6 km^2^, 23.1 km^2^, 19.3 km^2^, 17.6 km^2^, 12.0 km^2^ and 7.6 km^2^, respectively.

**Figure 4:**
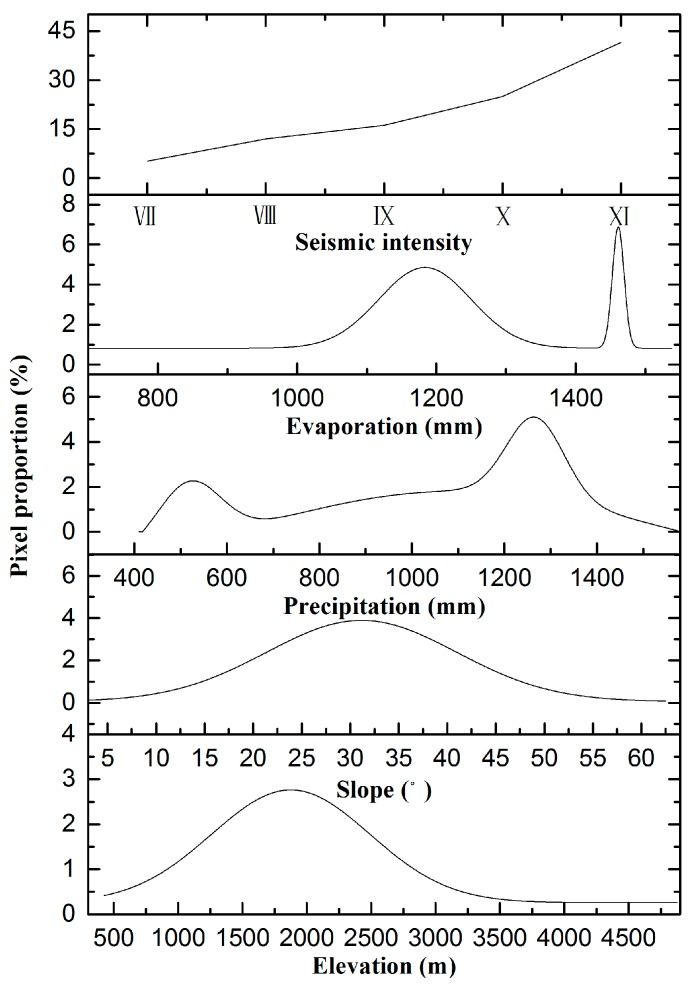
Distributions of damaged pixels.

The spatial distribution characteristics of the areas with vegetation damage were also revealed by distribution diagrams. The number of damage pixels varied with elevation, slope, annual precipitation, annual evaporation and seismic intensity (Figure 4). The damaged areas occurred primarily at elevations of 1000–3000 m and at slopes of 12.5°–47.5°, with an average elevation and slope of 1760 m and 30°, respectively. The peaks of the distribution curves for elevation and slope appeared at 1900 m and 32.5°, respectively. The damaged areas mainly occurred in hot dry valleys with 400–600 mm of precipitation and 1450–1500 mm of evaporation and in the humid region with 1000–1400 mm of precipitation and 1000–1300 mm of evaporation. The hot dry climate along Minjiang valley decreased the average precipitation value (1050 mm/a) of the whole damaged area and increased the average evaporation value (1210 mm/a). The distribution curves of precipitation and evaporation showed two peaks each at 525 mm and 1260 mm and at 1200 mm and 1475 mm, respectively. The distribution curve of seismic intensity showed that the damaged area expanded gradually as the intensity increased. The region that experienced an intensity of XI accounted for more than 40% of the entire damaged area. This observation indirectly demonstrated that higher earthquake intensities result in broader areas of vegetation damage. Regions with an elevation of 1900 m, a slope of 32.5°, an annual evaporation of 1475 mm and an annual precipitation of 1260 mm experienced the most vegetation destruction.

### 5.3. Fitting the Trend of Vegetation Recovery

**Figure 5:**
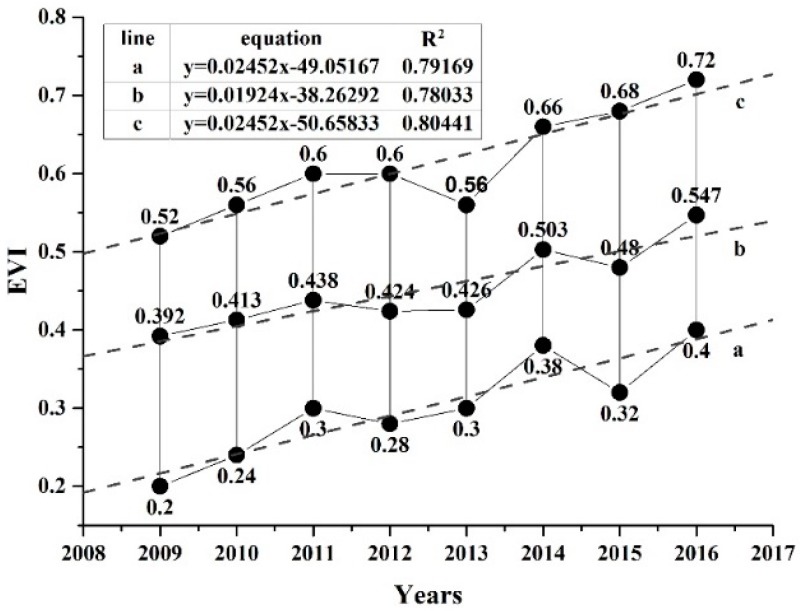
Statistical analysis of the most common components of EVI (Black solid line represents a large number of points; dotted lines a, b, and c are fitted to the minimum, mean and maximum EVI values of each year, respectively.).

**Figure 6:**
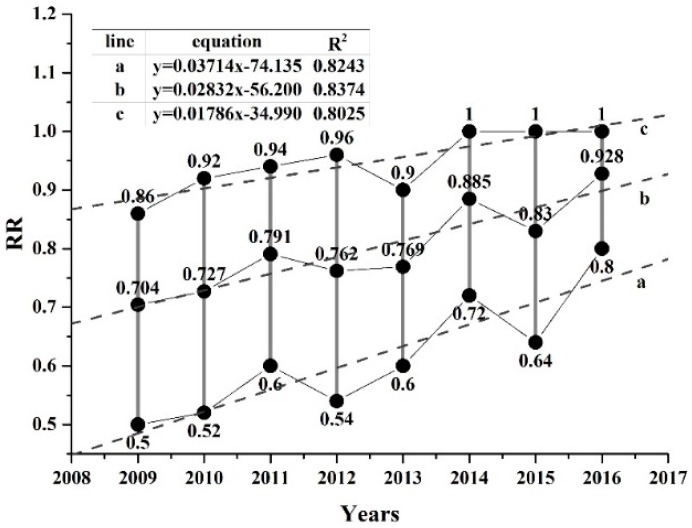
Statistical analysis of the most common components of RR (Black solid line represents a large number of points; dotted lines a, b, and c are fitted to the minimum, mean and maximum RR values of each year, respectively.).

The EVI distribution characteristics in each year post-earthquake were analysed by dividing the EVI value into 50 intervals with a range of 0.02 per interval. The most common EVI value ranges in 2009, 2010, 2011, 2012, 2013, 2014, 2015 and 2016 were 0.2–0.52, 0.24–0.56, 0.3–0.6, 0.28–0.6, 0.3–0.56, 0.38–0.66, 0.32–0.68, and 0.4–0.72, respectively, each of which accounted for nearly 80% of the total damaged pixels. Only the pixels included in the most common components of every year post-earthquake were identified and extracted as statistical samples, which were used to study the vegetation restoration trend. The total number of samples (the inter-annual coincident pixels) was 4923, accounting for 62.3% of the total damaged pixels. Then, we created a scatterplot of EVI versus the timing of each sample. The large number of points formed vertical lines for each year (black lines in Figure 5). Despite slight declines in some years, such as 2012, 2013, and 2015, the EVI values gradually increased with time overall (Figure 5). We fitted the minimum, mean and maximum EVI values of each year via linear regression, as shown in Figure 5. The coefficients of determination (R^2^) for the three equations were all approximately 0.8, indicating a strong linear recovery trend. Similarly, we selected 5112 statistical samples of RR, accounting for 64.7% of the total damaged pixels. The linear fitting curves are shown in Figure 6. The R^2^ values were approximately 0.82, slightly higher than those of the EVI, and also showed a clear linear recovery trend. We used RR to estimate the vegetation recovery trend, rather than EVI, because of the higher R^2^ values and the convenience of calculating the recovery time (i.e., the point at which the RR of all pixels returns to 1, in contrast to the point at which each pixel returns to some pre-earthquake EVI value).

### 5.4. Classification of Resilience 

**Figure 7:**
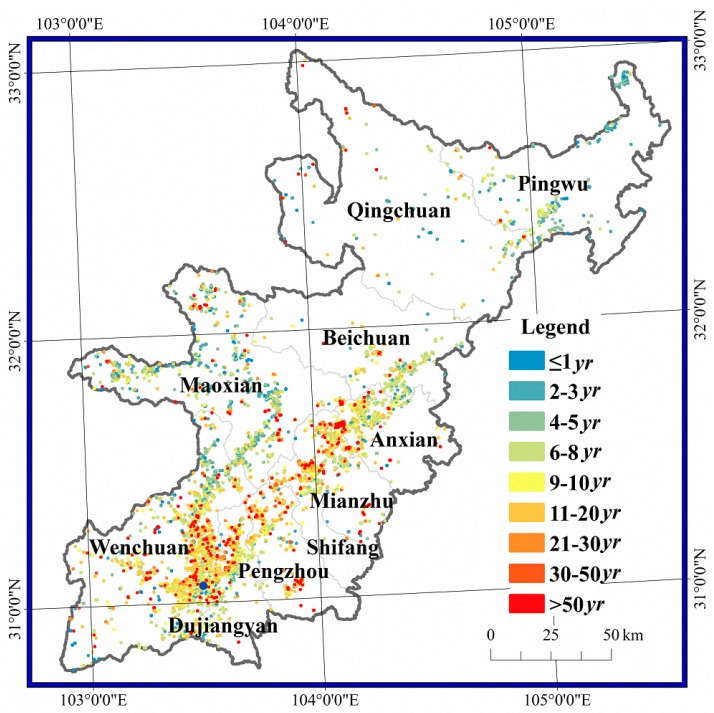
Spatial differences in recovery intervals and resilience levels.

Among the 7902 damaged pixels, 248 pixels were found to have negative *T* values (recovery time intervals), i.e., the vegetation would only recover if time reversed. The number of pixels with positive *T* values was 7654, and the vegetation in 7486 of these pixels is predicted to recover within 50 years. The lower the recovery time interval, the higher the resilience. The area with resilience values of 0< T≤50 accounted for 401.9 km^2^, representing 94.7% of the total damaged area, and had an average *T* value of 10 years. The area without resilience, i.e., T<0 or T>50, accounted for 22.2 km^2^, representing 5.3% of the total damaged area. The damaged pixels were further divided into 9 groups based on *T* values: “≤1 years”, “2–3 years”, “4–5 years”, “5–8 years”, “9–10 years”, “11–20 years”, “21–30 years”, “31–50 years” and “>50 years”. The pixel number, area and proportion of each group are shown in Table 1, and the spatial distribution is shown in Figure 7. In total, 44.19% of the damaged pixels reached or exceeded the pre-earthquake level. Among these pixels, 2.38% had an excellent resilience with a *T* of 1 year or less, 6.48% had a good resilience with a *T* of 2–3 years, and 35.33% had a moderate resilience with a *T* of 4–8 years. Furthermore, 36.38% of the damaged pixels required over 10 years to recover vegetation, 11.02% required over 20 years, and 5.26% required more than 50 years (very little resilience).

**Figure 8:**
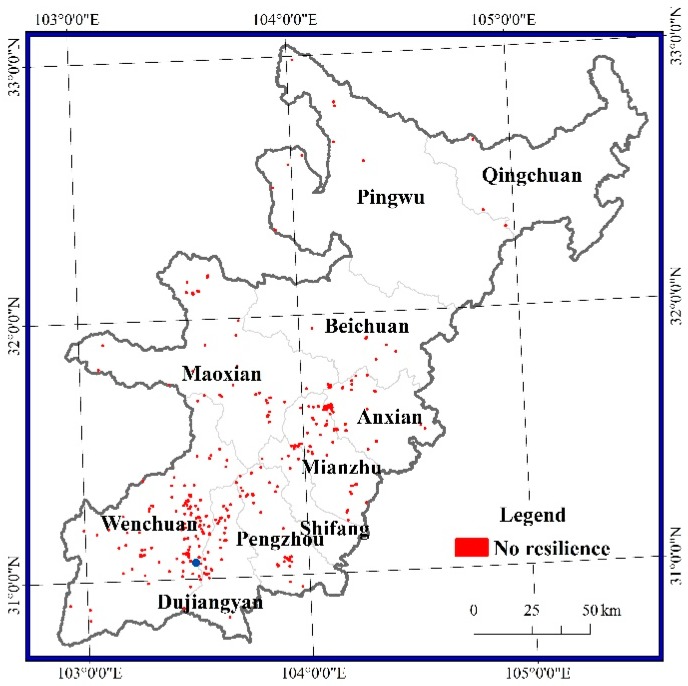
Spatial distribution of pixels without resilience.

The damaged pixels without resilience were scattered among the 10 counties but were mainly concentrated in the Yingxiu-Yinxing-Miansi section of the Minjiang River, Gaochuan village in Anxian, Qingping village in Mianzhu, Longmenshan town in Pengzhou, and Hongkou village and Longchi town in Dujiangyan (Figure 8). Most pixels without resilience were in Wenchuan and represented an area of 7.1 km^2^. The areas without resilience in Pingwu, Shifang, Beichuan and Qingchuan all accounted for less than 1 km^2^.

**Figure 9:**
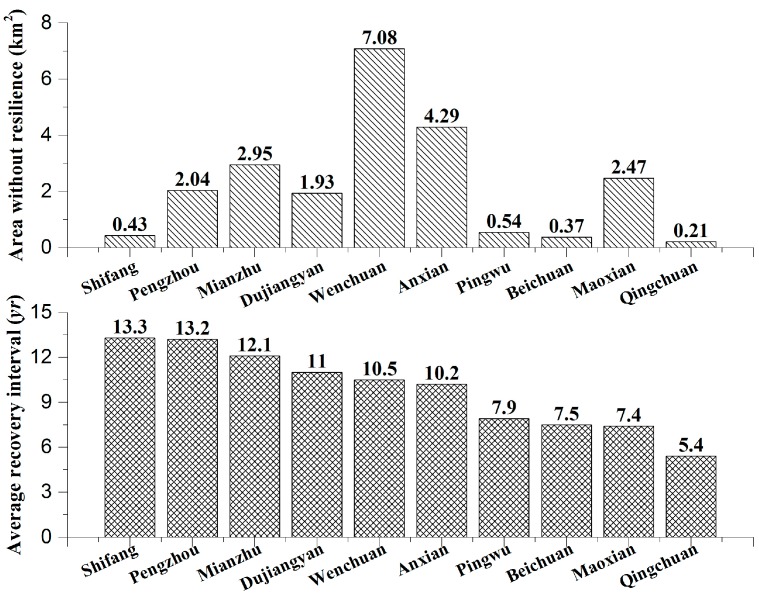
Comparisons of resilience among the 10 counties.

In Pengzhou, Mianzhu, Dujiangyan, Wenchuan and Anxian, the resilience levels of the damaged pixels were weak, with *T* values of greater than 10 years, and areas without resilience were relatively common. Despite a smaller area without resilience, the resilience of Shifang was also weak, with the longest average *T* of 13.3 years. The resilience levels of Pingwu, Beichuan, Maoxian and Qingchuan were higher, with *T* values of less than 8 years and relatively few areas without resilience (Figure 9).

### 5.5. Spatial Heterogeneity in Resilience and Its Influencing Factors

The resilience of the vegetation coverage was related to the topography, climate and substrate. In this study, the topography was characterized by elevation and slope; the climate was characterized by annual precipitation and evaporation; and the substrate was characterized by land use, soil and lithology. The average recovery interval was calculated for different classes of topography, climate and substrate conditions, and the relationships among resilience and these various factors were analysed.

**Figure 10:**
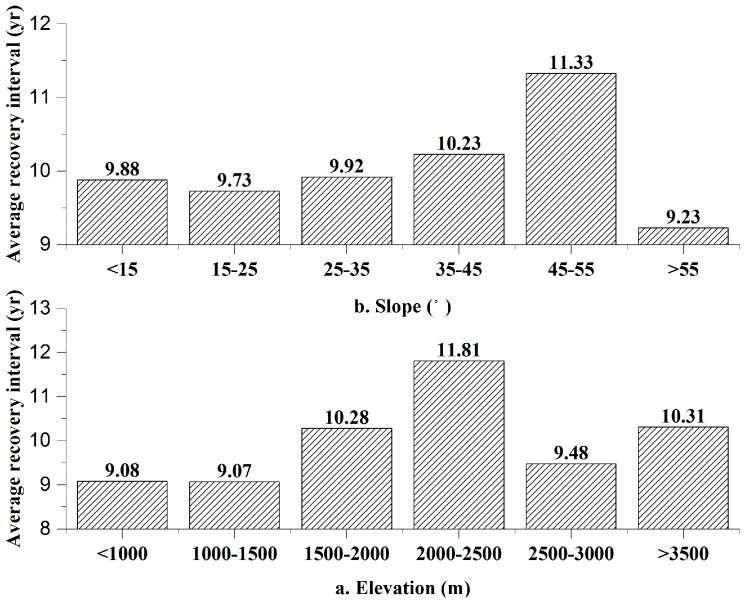
Relationships among resilience and topographic factors.

The relationship between recovery interval and elevation is shown in Figure 10a. At elevations of less than 2000 m, as the elevation increased, the average *T* increased, and the resilience decreased gradually. From 2000–2500 m, the recovery interval was the longest, and the resilience was the lowest. The areas with the lowest resilience were 100–600 m higher than the average elevation of the damaged areas, i.e., 1900 m. Above 2500 m, the recovery interval decreased, and the resilience slightly increased. The relationship between *T* and slope is shown in Figure 10b. For slopes of less than 55°, as the slope increased, the average *T* increased, and the resilience decreased. Above 55°, the resilience rebounded slightly. Based on the average slope of the damaged areas of 32.5° and the fact that the vast majority of the damaged areas had slopes of less than 55°, we concluded that the resilience was inversely proportional to slope.

**Figure 11:**
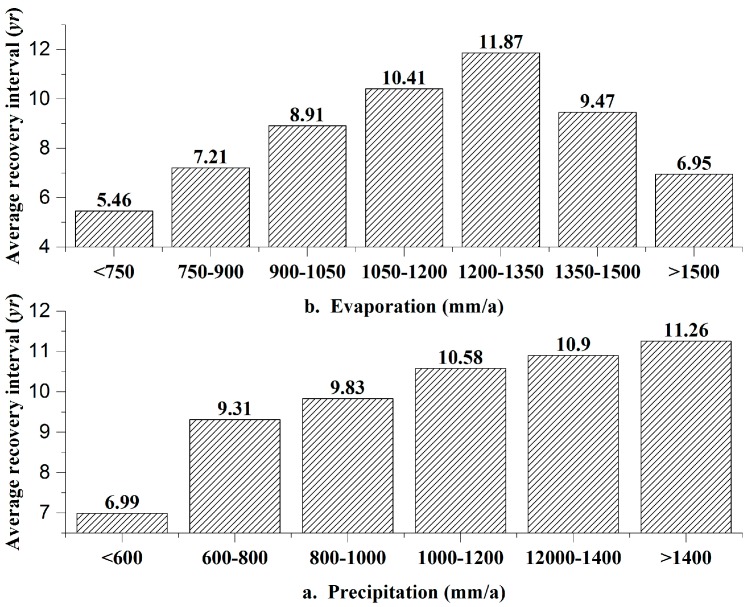
Relationships among resilience and climate factors.

The relationship between recovery interval and annual precipitation is shown in Figure 11a. As the precipitation increased, the average *T* increased, and the resilience decreased. The resilience was obviously inversely proportional to precipitation.

**Figure 12:**
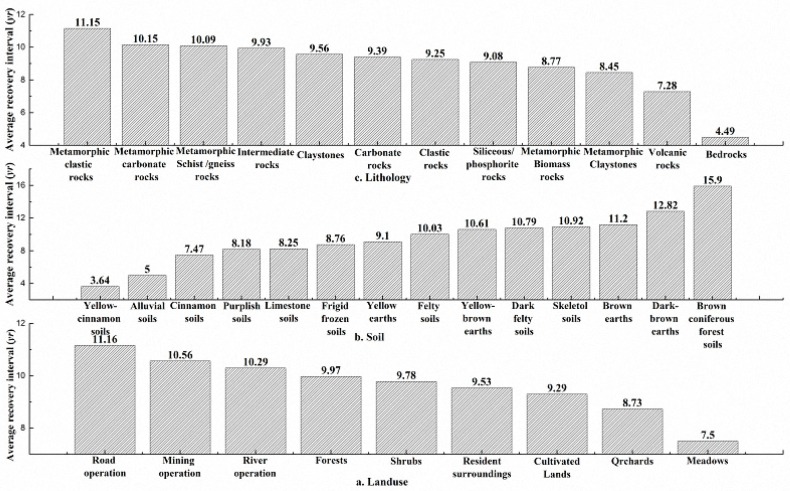
Relationships among resilience and substrate factors.

However, this was not the case in the relationship between recovery interval and annual evaporation, as shown in Figure 11b. For evaporation values less than 1350 mm, the resilience decreased with increasing evaporation, whereas for evaporation values greater than 1350 mm, the resilience increased slightly with increasing evaporation. The relationship between the recovery interval and land use is shown in Figure 12a. EVI values are synthetic values based on vegetation mixed with the surroundings, such as roads, rivers and mines, due to the relatively low resolution (250 m) of this product. A pixel was defined as a road, river or mining operation when it was crossed by a road or river or contained part of a mining area. Meadow areas had the shortest recovery interval and the highest resilience. The resilience was good in areas with frequent human activity, such as arable land, gardens and residential areas. Forest had a weak resilience. Roads, rivers, and mining operations had the worst resilience levels, with *T* values of greater than 10 years. The relationship between recovery interval and soil is shown in Figure 12b. The recovery interval of brown coniferous forest soil was 15.9 years, representing the lowest resilience, whereas the recovery interval of yellow cinnamon soil was 3.6 years, representing the best resilience. The relationship between recovery interval and lithology is shown in Figure 12c. The resilience of areas underlain by metamorphic rock, especially metamorphic clastic rock, was the weakest, with *T* values of 11.2 years. The resilience of areas underlain by sedimentary rock was better, and the resilience of areas underlain by igneous bedrock was the best, with a recovery interval as short as 4.5 years.

The resilience of the vegetation coverage was obviously related to topographic, climatic and substrate factors. The resilience was inversely related to the steepness of the terrain and the interference intensity of climatic factors and positively related to the stability of the substrate. The resilience of meadow areas was better than that of shrub areas and much better than that of woodland areas. Similarly, the resilience of residential areas was higher than that of areas with roads, rivers, or mining operations.

## 6. Discussion

### 6.1. Validation of the Damaged Area Estimate

**Figure 13:**
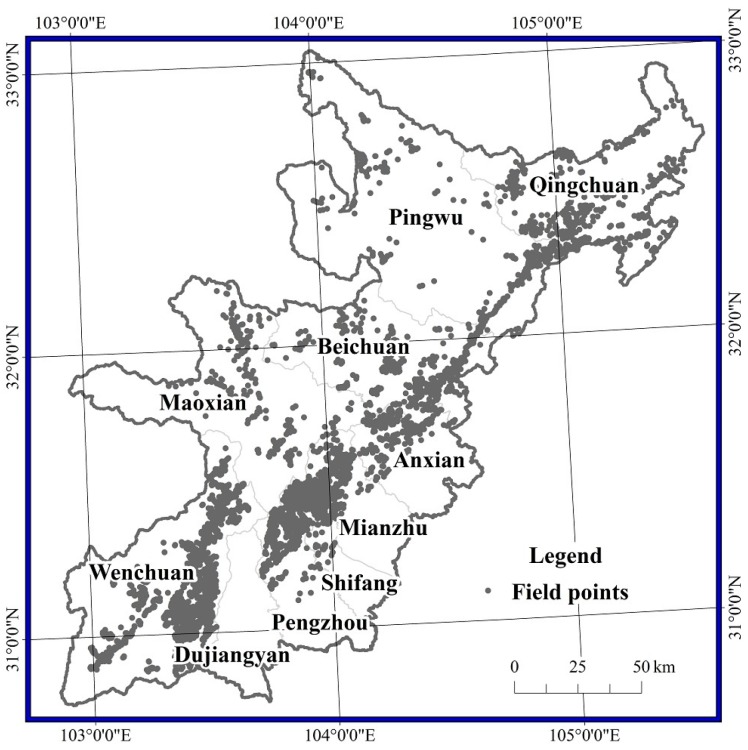
Distribution of field survey points.

To validate the extraction of damaged vegetation, we collected 4751 field survey points from the Ministry of Land and Resources of China, including collapses, landslides, debris flows and other geological disasters, which were identified by geological experts soon after the earthquake (Figure 13). A total of 3441 points (72.4% of all the field points) fell within the damaged areas. The remaining 1310 points did not fall in the damaged areas, possibly because the resolution of the MODIS data is too low to capture small damaged patches or because some pixels affected by the earthquake were not damaged enough to be identified. Jiang et al. performed a similar verification and found that 2950 points (62.09%) fell within the damaged area he extracted [35]. Notably, the damaged area in Jiang’s work covered 4756.9 km^2^, almost ten times greater than the estimate in this research (424.1 km^2^). The following reasons may explain the large difference. Firstly, Jiang et al. used MODIS-NDVI data and set the vegetation damage rate (VDR) to VDR ≥ 13.47% as the standard for damaged extraction (VDR = (NDVI_1_ − NDVI_0_)/NDVI_0_, where NDVI_0_ and NDVI_1_ represent the vegetation coverage pre-earthquake and immediately post-earthquake, respectively). The NDVI values are easily saturated due to the high vegetation coverage in the study area; thus, the threshold VDR = 13.47% may be too low. Lu et al. used the same method to extract the damaged area using a threshold of VDR = 30% [31]. In this research, we did not set a uniform threshold for all pixels and instead set independent thresholds for each pixel by calculating the maximum natural fluctuation. The maximum, minimum and mean VDR values were 100%, 17.3% and 45.4%, respectively, in our work. The minimum VDR exceeded the threshold of Jiang et al. Thus, we infer that the damaged area extracted by Jiang et al. contained many areas with natural declines in vegetation. This was also indirectly demonstrated by Chong et al., who extracted a total landslide area of 1150.62 km^2^ across a total area of 44,031 km^2^ using multi-source high-resolution images [43,44].

**Figure 14:**
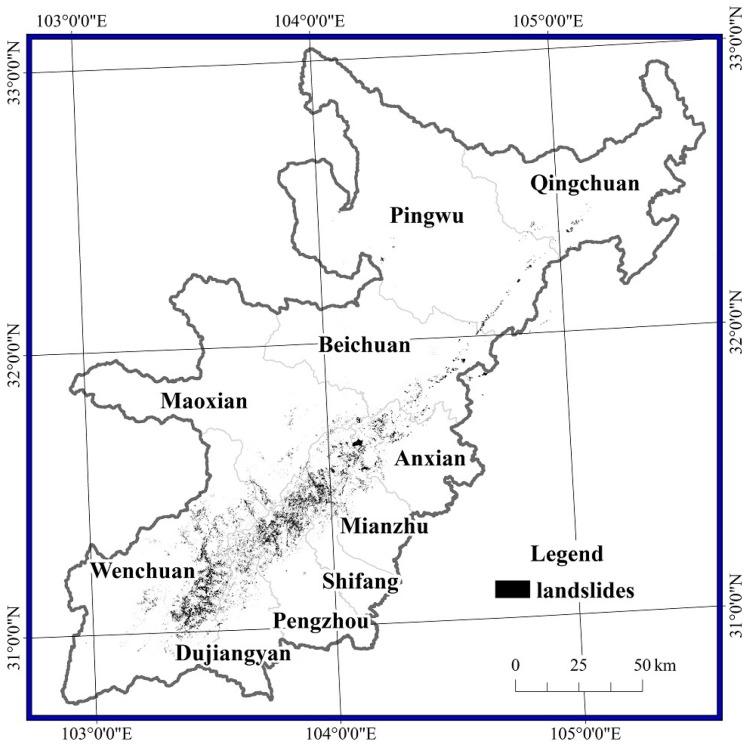
Eroded areas extracted from Landsat images.

**Figure 15:**
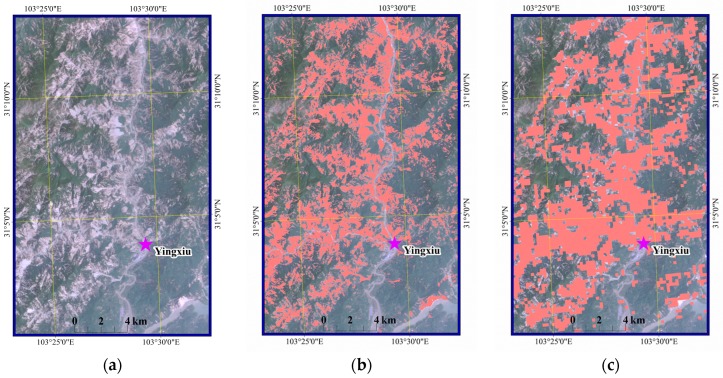
Damaged area extraction and comparison, (**a**) Landsat TM image; (**b**) the eroded area extracted from the TM image; (**c**) the damaged area extracted from the MODIS data.

We also found that a total area of 489.4 km^2^ experienced erosion based on Landsat images before and after the earthquake (Figure 14). In total, 52.7% of the eroded area was included in the damaged area extracted from the MODIS-EVI data. Another 25.4% of the eroded area lay within 500 m of a damaged pixel (a distance equivalent to almost two MODIS pixels). The remaining eroded area was far from the damaged pixels. These differences may be due to both the resolutions and the geographic coordinate projection error between the MODIS and TM products. A high-resolution TM image of the vicinity of the epicentre in Yingxiu is shown in Figure 15a. The distributions of eroded and damaged areas are shown in Figure 15b,c, respectively. Most large damaged areas were extracted, while some fragmented and scattered areas were not identified.

### 6.2. Resilience Comparison

The damaged vegetation area occurred at elevations between 1000 m and 3000 m and on slopes between 12.5° and 47.5°. These ranges were basically consistent with Chong’s research (1000–3000 m, 15°–55°) [44] but differed slightly from Lu’s ranges (1500–2500 m, 25–55°) [31] and Zhang’s ranges (1000–2250 m, 25°–55°) [45] because the latter studies focused on particular regions, such as Maoxian, the Mianyuan River and the Subao River basin. Higher seismic intensities resulted in more severe damage, similar to the findings of Chong’s study [44].

Yang and Qi grouped the post-seismic vegetation conditions into three major classes: deteriorating, fluctuating, and recovering, which were characterized by positive, zero, and negative values of the ED_NDVI_ changing rate, respectively [42]. Despite the difference in the absolute area of damaged vegetation, the proportions of each group may be similar to the results of our work. Pixels without resilience (*T* < 0) accounted for 3.13% of the entire damaged area, close to the deteriorating proportion (4%) derived by Yang and Qi [42]. Pixels with a certain degree of resilience (0 < *T* ≤ 10) accounted for 63.62% of the entire damaged area, slightly different from the recovery proportion (59%) derived by Yang and Qi [42]. Pixels with poor resilience (*T* > 10, with (slopes approaching zero) accounted for 33.52% of the whole damaged area, also slightly different from fluctuating proportion (37%) derived by Yang and Qi [42].

The resilience was better at lower and higher elevations and poorer at intermediate elevations, which was also observed in Jiang’s research [35]. Meadow vegetation featured the highest resilience and recovered rapidly, which has also been confirmed by Jiang [35]. However, Yang et al. found that grassland has the smallest recovering percentage compared to shrub, needleleaf and mixed vegetation. The difference could be caused by the distinct spatial scales of land use between our work and that of Yang and Qi. The resilience was inversely related to the precipitation, probably because of the geological disaster vulnerability in the study area. Heavy precipitation easily triggered landslides and debris flows, thereby accelerating the ecological degradation. A similar conclusion was reached in Wang’s study [33]. Notably, the persistence of loose material increased the risk of rainfall-triggered landslides and debris flows [33]. The resilience in hot valleys was not the lowest, but the ecological restoration was still not ideal, as the natural restoration process was slow in the dry hot valleys [31]. The resilience of yellow cinnamon soil was the highest, partly because of its high clay content and low susceptibility to soil erosion. Similar results can be found in Jiang’s research. Areas with higher clay contents were associated with smaller areas of non-recovered vegetation.

### 6.3. Innovation

We attempted to use MODIS-EVI data to study the vegetation damage and recovery process. Compared with use of MODIS-NDVI data in previous studies, MODIS-EVI data produced a stronger response to the ecological impacts of the earthquake. Saturated NDVI values produce defective vegetation trends in areas with high vegetation coverage. In this research, the damaged vegetation area was extracted by setting independent thresholds for each pixel, rather than setting a uniform threshold for all pixels, as performed in previous studies. Although the damaged area was smaller than that in Jiang’s research, the accuracy was higher. The innovation of this study is the integration of the resilience concept into a post-earthquake ecological assessment. This integration is helpful not only in estimating the current situation of ecological restoration but also in predicting the ecological evolution trend. We defined resilience as the recovery time. Based on the statistical information extracted from the most common components, the resilience was calculated by fitting an equation to the RR versus time. We determined whether a pixel will ever be able to recover to the pre-earthquake level and the recovery time required in the future. We also analysed the reasons for the spatial differences. This information is relevant to scientific management and spatial planning of the damaged ecosystem.

### 6.4. Uncertainty

In this research, we extracted the damaged area by setting the maximum pre-earthquake natural variation as the threshold. In some pixels, the EVI value decreased due to the earthquake but did not exceed the threshold; thus, these pixels were not included in the damaged area. This loss indicates that this method could be improved. A linear method was used to fit the ecological restoration trend based on the statistical characteristics of the most common components. The most common components accounted for 64.7% of the total damaged pixels. We had no adequate information to determine whether the remaining 35.3% of the damaged pixels have a linear trend. Eight values between 2009 and 2016 are enough to implement linear fitting for most pixels (the vegetation in 80% of the pixels will fully recover by 2022), especially pixels that will experience complete vegetation recovery soon. However, for pixels that had a long recovery time, 8 sample values are not sufficient for fitting. More annual data in the future should be collected to validate the vegetation trends and linear fitting results.

## 7. Conclusions

The 2008 Wenchuan earthquake severely damaged the ecosystem. The damaged area was extracted using MODIS-EVI data based on the obvious post-earthquake decline in vegetation coverage. A total number of 7902 pixels (424.1 km^2^) were extracted as the damaged area from a total area of 26,000 km^2^. In the damaged areas, the vegetation decreased by 45.4%, with greater reductions occurring in areas that experienced greater shaking. The damaged areas were mainly distributed along the Min River, Yuxi River and Longmenshan fault and had an average elevation of 1760 m, a slope of 30°, an annual precipitation of 1050 mm and an annual evaporation of 1210 mm. Great changes have occurred in the disaster area over the subsequent 8 years, and research on vegetation recovery and vegetation evolution prediction in the future remains important for disaster prevention and mitigation.

By 2016, the vegetation in 44.2% of the damaged area had recovered to pre-earthquake levels, and the vegetation in 50.5% of the damaged will require more than 8 years but less than 50 years to fully recover, with an average recovery interval of 13.7 years. Thus, the majority of the damaged area will have recovered by 2022. However, 5.3% of the damaged had almost no resilience, exhibiting recovery intervals of greater than 50 years, and these areas should receive more attention in the future.

The resilience was closely related to the site conditions. The resilience was negatively correlated with slope and annual precipitation but exhibited an inverted parabolic shape with elevation and annual evaporation. The lowest resilience value appeared at elevations of 2000–2500 m and annual evaporation levels of 1200–1350 mm. The vegetation recovered rapidly in meadows but more slowly in woodlands. Areas with roads, rivers and mining operations were also slower to recover. Stable bedrock improved vegetation recovery, resulting in a high resilience, while unstable metamorphic rock hindered vegetation recovery, resulting in a poor resilience. We should pay more attention to the lithologically unstable areas at elevations of 2000–2500 m, slopes of 30°, precipitation levels of 1200 mm and evaporation levels of 1300 mm, especially near roads, rivers and mining operations. Artificial reconstruction or other direct measures should be considered to prevent geological hazards and ensure the safety of people.

## Figures and Tables

**Table 1:** The resilience of the damaged pixels.

Classification of Resilience	Recovery Interval	Number of Pixels	Area (km^2^)	Percentage of the Total Damaged Area
Excellent	≤1 year	188	10.09	2.38%
Good	2–3 years	512	27.48	6.48%
Moderate	4–5 years	376	20.18	4.76%
	6–8 years	2416	129.65	30.57%
Low	9–10 years	1535	82.38	19.43%
	11–20 years	2004	107.54	25.36%
Bad	21–30 years	301	16.15	3.81%
	31–50 years	154	8.26	1.95%
No resilience	>50 years	416	22.32	5.26%
